# The effects of the Xijiao Dihuang decoction combined with Yinqiao powder on miRNA-mRNA profiles in mice infected with influenza a virus

**DOI:** 10.1186/s12906-020-03074-4

**Published:** 2020-09-21

**Authors:** Ke Li, Xiaoming Chen, Jing Zhong, Hehe Ye, Shujing Zhang, Dongyu Ge, Xudan Wang, Ying Wu

**Affiliations:** 1 Department of Microbiology and Immunology, School of Life Sciences, Beijing University of Chinese Medicine, Beijing, 102400 China; 2 Center of Research and Experiments, Institute of Traditional Chinese Medicine, Beijing University of Chinese Medicine, Beijing, 102400 China; 3 Clinical Medicine Research Centre, Liuzhou People’s Hospital, Guangxi Medical University, Liuzhou, 545001 China

**Keywords:** Influenza a virus (IAV), Pneumonia, miRNA, mRNA, Xijiao Dihuang decoction combined with Yinqiao powder (XDY)

## Abstract

**Background:**

MicroRNAs (miRNAs) play vital roles in acute inflammatory and antiviral responses during influenza A virus (IAV) infection. The Xijiao Dihuang decoction combined with Yinqiao powder (XDY) is applied to remedy viral pneumonia in China and its therapeutic efficacy in pneumonic mice challenged with IAV was demonstrated; however, the underlying mechanisms remain elusive. Thus, this study aimed to explore the miRNA-mRNA profiles in the lungs of IAV-infected mice and investigate the therapeutic mechanisms of XDY involving miRNAs and associated pathways.

**Methods:**

We detected the cellular miRNA contents in the lungs of mice treated with XDY (23 g/kg/d) for A/FM/1/47 (H1N1) (FM1) infection at 4 days postinoculation (dpi) and 7 dpi. MiRNA and mRNA high-throughput sequencing analyses, and miRNA and mRNA qRT-PCR analyses were used to detect and verify the relevant miRNAs and mRNAs. Conjoint analysis, GO enrichment analysis, and KEGG database analysis were applied to identify the miRNA-mRNA regulatory relationships.

**Results:**

The quantities of differentially expressed miRNAs and mRNAs were upregulated over time. The data showed that 104 miRNAs and 3485 mRNAs were differentially expressed after challenge with FM1 on day 4, while 191 miRNAs and 6126 mRNAs were differentially expressed on day 7. The GO enrichment analysis and KEGG database data showed that the differentially expressed miRNAs and mRNAs were mainly enriched in JNK activity, MAPK phosphatase activity, and the TLR, Jak-STAT and TNF signalling pathways after treatment of FM1 infection with XDY. Generally, the expression trends of differentially expressed miRNAs and mRNAs based on the qRT-PCR results exhibited good consistency with the results of the high-throughput sequencing analysis.

**Conclusions:**

MiRNAs and mRNAs were differentially expressed during FM1 infection. The therapeutic mechanisms of XDY in FM1-infected mice, might be related to regulating antiviral immunity and ameliorating excessive inflammatory responses by modulating the expression of dysregulated miRNAs and mRNAs involved in the ERK/JNK-AP-1, and IFN-β/STAT signalling pathways.

## Background

The influenza A virus (IAV) belongs to the family Orthomyxoviridae and poses a severe threat to human health. Within the past 100 years, the influenza A H1N1, H2N2 and H3N2 viruses have caused pandemics and resulted in high morbidity and mortality worldwide [1]. By activating innate and adaptive immunity, IAV triggers acute inflammatory responses, including widespread epithelial damage, multifocal destruction, desquamation of the trachea, vascular leakage and lung oedema [1–3]. These inflammatory responses are associated with the expression of immune response-related genes [4].

MicroRNAs (miRNAs) represent a class of 19–24 nucleotide (nt) noncoding RNA molecules that can downregulate gene expression posttranscriptionally by cleaving mRNAs or repressing protein translation via sequence complementarity between miRNAs and their target mRNAs [5, 6]. MiRNAs serve to regulate biological processes, including development, gene transcription, translation, differentiation and apoptosis [7, 8]. Therefore, it comes as no surprise that miRNAs have a substantial influence on IAV infection and pathogenesis. Many studies have focused on the roles of differentially expressed miRNAs in IAV-infected cells or tissues from animal hosts, such as mice or pigs. In a study by Moran, the results showed that the high expression of miR-150 accelerated the production of pro-inflammatory cytokines in A/H1N1/pdm09-infected A549 epithelial cells [9]. Wu et al. reported that the TGF-β pathway might be involved in the pathophysiological process underlying A/PuertoRico/8/34 (H1N1) (PR8) and A/Beijing/501/2009 (H1N1) (BJ501) infection and was activated via the upregulation of miR-223 and miR-155 in the lungs of mouse models [10].

Several major studies indicated that the expression of miRNAs such as miR-34b and miR-34c-3p were temporal and virus strain-specific and showed no remarkable change during A/chicken/Germany/R28/2003 (H7N7) infection, whereas such miRNAs were markedly upregulated during infection with A/Mexico/InDRE4487/2009 (H1N1) [11]. Significant downregulation of let-7f was found in PR8-infected rather than A/Moscow/10/99 (H3N2)-infected human lung epithelial cells (A549) [12]. Moreover, miR-139-5p, miR-27a-5p, miR-29b-1-5p, and miR-877-3p were upregulated during A/Aquatic bird/Korea/ma81/2007 (H5N2) (ma81) infection but not A/Aquatic bird/Korea/w81/2005 (H5N2) (w81) infection [13]. Li et al. identified 67 differentially expressed cellular miRNAs between infection with the H5N1 avian influenza virus (HPAI) and the reconstructed 1918 H1N1 influenza virus (r1918) (2:6) [14]. Influenza A/FM/1/47 (H1N1)(FM1), is commonly used by Chinese researchers to investigate the immune response and inflammatory pathogenesis during IAV infection, and to evaluate the therapeutic and pharmacological effects of Chinese herbs [15–21]. However, the FM1-induced general changes in the levels of miRNAs and mRNAs remain unclear. In terms of the strain-specific and temporally-dependent signatures of miRNA expression, we profiled the global patterns of miRNAs and mRNAs in the lungs of FM1-infected mice.

Xijiao Dihuang decoction combined with Yinqiao powder (XDY) is a classical compound prescription of traditional Chinese medicine derived from *Wenbing Tiaobian*, which was first created by Wu, Jutong in the Qing Dynasty. Xijiao Dihuang decoction, which contains dried rehmannia root (roots of *Rehmannia glutinosa (Gaertn)Libosch*), Chinese herbaceous peony (roots of *Paeonia lactiflora Pall*), moutan bark (root barks of *Paeonia suffruticosa Andr.*) and buffalo horn (horns of *Bubalus bubalis Linnaeu*), is recommended to patients who suffer from pneumonia, alimentary tract haemorrhage, radiation enteritis and sepsis. Recent studies indicated that Xijiao Dihuang decoction (XJDHD) had protective effects on lipopolysaccharide (LPS) and tumour factor alpha (TNF-α)-induced acute liver failure in mice [22–24]. Li et al. reported that XJDHD could cure viral pneumonia by nourishing yin, reducing fever, dispelling phlegm and cooling the blood [25]. Yinqiao powder, which is composed of honeysuckle (flower buds of *Lonicera japonica Thunb.*), Forsythiae Fructus (Fructus of *Forsythia suspensa (Thunb.) Vahl*), Balloon Flower root (roots of *Platycodon grandiflorum*), mint (whole *Mentha haplocalyx Briq.* plants), fermented soybean (fermented products of *Glycine max (L.)Merr.*), herba lophatheri (leaves of *Lophatherum gracile Brongn*), great burdock achene (Fructus of *Arctium lappa L.*), schizonepeta spike (spicas of *Schizonepeta tenuifolia Briq.*) and liquorice root (roots of *Glycyrrhiza uralensis Fisch.*), is recommended to be consumed by patients who suffer from upper or lower respiratory tract infection, mumps, viral myocarditis, and epidemic haemorrhagic fever. Clinical studies demonstrated that Yinqiao powder had anti-inflammatory and antiviral effects on patients infected with H1N1. Recent studies suggested that Yinqiao powder might inhibit viral replication, alleviate excessive inflammatory reactions, boost immunity and improve blood circulation during H1N1 infection by modulating the Toll-like receptor (TLR) 7/nuclear factor kappa B (NF- κB) pathway [26]. As elaborated by Wu, Jutong in *Wenbing Tiaobian*, by alleviating epidemic heat syndrome with Yinqiao powder and clearing the latent heat of the blood system with XJDHD, XDY shows enhanced efficacy compared to XJDHD or Yinqiao powder for the therapy of epidemic febrile diseases [27]. In mice infected with FM1, XDY was demonstrated to have therapeutic effects on viral pneumonia by reducing viral titres, improving the lung index, ameliorating oedema and pulmonary vascular permeability, and alleviating other clinical manifestations. In addition, in contrast to a low dose (11.5 g/kg· d) or a high dose (46 g/kg· d), the clinical equivalent dose (23 g/kg · d) of XDY exhibited the most dramatic protective effects on pneumonic mice [28]. Moreover, XDY attenuated pulmonary gross lesions and tissue pathological changes more effectively than Yinqiao powder or XJDHD in FM1-infected pneumonic mice [29]. Regarding the therapeutic mechanisms in IAV-infected mice, XDY might prevent activation of the inflammatory cascade by inhibiting the formation of the nucleotide oligomerization domain (NOD)- like receptor pyrin domain-containing 3 (NLRP3) inflammasome, reducing the secretion of interleukin (IL)-1β and IL-18, and downregulating the expression of intercellular adhesion molecule-1 (ICAM-1) and vascular cell adhesion molecule-1 (VCAM-1). Moreover, XDY was suggested to improve hyperpermeability in viral pneumonia by downregulating the G protein coupled receptor kinase 2 (GRK2) protein, and upregulating β-adrenergic receptors (β-AR) and G protein (GS) α [30–32]. In vitro, XDY also showed anti-inflammatory effects in alveolar macrophages (NR8383) infected with FM1 by repressing the production of TNF-α, and monocyte chemoattractant protein-1 (MCP-1), as well as by reducing the concentration of prostaglandin E-1 (PGE1), phospholipase A2 (PLA2), leukotriene B4 (LTB4) and oxygen free radicals [33]. XDY could alleviate hyperpermeability and F-actin reorganization induced by FM1 in primary pulmonary microvascular endothelial cells (PMVECs) by repressing the phosphorylation of ezrin/radixin/moesin (p-ERM) via inhibition of the Rho/Rho kinase 1 (ROCK), p38 MAP kinase (MAPK), and protein kinase C (PKC) pathways [34]. Nevertheless, the comprehensive demonstration of the therapeutic mechanisms of XDY is still lacking and needs to be further explored. Thus, we preliminarily examined the effects of XDY on miRNA-mRNA profiles and associated signalling pathways in lung tissues of FM1-infected mice to shed new light on the therapeutic mechanism of XDY in IAV-induced viral pneumonia.

## Methods

### Chemicals and reagents

Anaesthetic ether, xylene, chloroform and formalin were purchased from Beijing Chemical Works (Beijing, China). Ethanol was obtained from Shandong LIRCON Medical Technology Co., Ltd. TRIzol RNA isolation reagent was purchased from Invitrogen (Carlsbad, USA). miScript HiSpec Buffer, miScript Nucleics Acid Mix, miScript Reverse Transcriptase Mix, RNase-free water oligo primer, reaction buffer, dNTPs, revertase, and RNase inhibitor were obtained from Qiagen (Dusseldorf, Germany).

### Virus

Influenza virus A (FM1/1/47 strain) (FM1), a murine lung-adapted strain, was provided by the Institute of Microbiology, Chinese Academy of Sciences. The virus was propagated in the allantoic cavities of 9-day-old SPF embryonic chicken eggs (Beijing Boehringer Ingelheim Vital Biotechnology Co., Ltd.) at 37 °C for 48 h followed by preservation at − 20 °C for 1 h and transfer at 4 °C overnight. The virus-containing allantoic fluid was harvested and the viral titre was detected. Allantoic fluid was serially diluted 10- fold (2^0^–2^− 11^) with sterile phosphate buffered solution at 200 μl/well in a parallel 24- well plate (Thermo). Two hundred microlitres of 0.2% (V/V) chicken erythrocyte suspension was mixed with diluted allantoic fluid in each well. The mixture was incubated at 37 °C (Thermo) for 1 h. The viral titre was calculated by implementing the Reed-Muench method and the TCID_50_ was estimated to be 2^3.8^ per μl. Finally, the allantoic fluid was stored at − 80 °C until use.

### Preparation of the XDY decoction

The components of XDY were purchased from Beijing Tongrentang Pharmaceutical Co. Ltd., China. XDY was made up of 13 herbs, including dried rehmannia root (30 g), buffalo horn (30 g), Chinese herbaceous peony (12 g), moutan bark (9 g), honeysuckle (9 g), Forsythiae Fructus (9 g), Balloon Flower root (9 g), mint (6 g), Fermented Soybean (5 g), Herba lophatheri (4 g), Great Burdock Achene (9 g), Schizonepeta spike (5 g) and liquorice root (5 g)**.** The details of the XDY ingredients are listed in Table 1. In this paper, the administered dose of XDY for mice was determined as follows:

$$ \mathrm{BSA}=\mathrm{k}\ast {\mathrm{mass}}^{0.667} $$

where BSA is the body surface area of the mice, the mass is the body mass of the mice, and k is the Meeh constant, which is equal to the dose conversion factor (F) of the mouse and adult human. The k of the normal mouse and adult human is approximately 10 [35].

$$ {\mathrm{D}}_{\mathrm{m}}={\mathrm{D}}_{\mathrm{h}}\times \mathrm{F}/\mathrm{W} $$

where D_h_ is the clinical dose of XDY for adult humans, D_m_ is the administered dose of XDY for mice, F is the dose conversion factor for mice and adult humans, and W is the weight of the adult human body [36]. D_h_ was 142 g, (dried rehmannia root 30 g, buffalo horn 30 g, Chinese herbaceous peony 12 g, moutan bark 9 g, honeysuckle 9 g, Forsythiae Fructus 9 g, Balloon Flower root 9 g, mint 6 g, Fermented Soybean 5 g, Herba lophatheri 4 g, Great Burdock Achene 9 g, schizonepeta spike 5 g and liquorice root 5 g). W was set to 60 kg, and F was 10. A D_m_ of 23 g/kg was calculated in our study.

**Table 1:** Composition of Xijiao Dihuang Decoction and Yinqiao Powder (XDY)

Decoction	English name	Latin name	Part used	Amount (g)
Xijiao Dihuang Decoction	Buffalo horn	*Bubalus bubalis* Linnaeu	horn	30
	dried rehmannia root	Rehmannia Glutinosa (Gaertn.) Libosch.	root	30
	Chinese herbaceous peony	*Paeonia lactiflora* Pall root	root	12
	moutan bark	*Paeonia suffruticosa* Andr.	Root bark	9
Yinqiao Powder	honeysuckle	*Lonicera japonica* Thunb.	Flower bud	9
	Forsythiae Fructus	*Forsythia suspensa* (Thunb.) Vahl	Fructus	9
	Balloon Flower root	*Platycodon grandiflorum* (Jacq.) A. DC.	root	6
	Mint	Mentha haplocalyx Briq.	Whole plant	6
	Licorice root	Glycyrrhiza Uralensis Fisch.	Root	5
	Herba lophatheri	Lophatherum Gracile Brongn	leaf	4
	Fermented Soybean	*Glycine max* (L.) Merr.	Fermented product	5
	Schizonepeta Spike	Schizonepeta tenuifolia Briq.	Spica	5
	Great Burdock Achene	*Arctium lappa* L.	Fructus	9

Briefly, all herbs were soaked in distilled water for approximately 1 h, and the buffalo horn was preboiled for approximately. After that, the rest of the herbs except mint were boiled with buffalo horn until 15 min after ebullition. A half-quantity of mint was added into the mixture and boiled for 5 min. The liquid was filtered, and the medicinal materials were secondarily boiled secondary until 15 min after ebullition. The rest of the mint was added to the mixture and boiled for 5 min. The supernatant filtered from the primary and secondary procedures was mixed and condensed to a total volume of 92.6 ml (1.5 g/ml). Finally, the decoction was preserved at 4 °C.

### Animals and treatment

Healthy specific pathogen-free (SPF) male BALB/C mice (16–20 g) were provided by Beijing Vital River Laboratory Animal Technology Company (license number: scxk 2012–0001). The animals were raised in an SPF laboratory animal room under standard temperature and humidity conditions and a 12 h light–dark cycle with access to water and food ad libitum.

A total of 30 mice were divided randomly into the control, virus and XDY groups (*n* = 10). After they were anaesthetized with ether, the mice in the virus and XDY groups were intranasally inoculated with 25 μl of a 30-fold dilution of LD_50_ FM1 in PBS, while the mice in the control group were administered an equivalent volume of normal saline. One hour after infection, the FM1-infected mice in the XDY group were intragastrically administered 0.15 ml XDY, twice a day, for 4 or 7 days, while the mice in the control group and virus group were intragastrically administered double distilled water. The fur and vitality of the mice were observed and recorded every day. Mice were weighed daily before drug delivery.

### Haematoxylin and eosin (H&E) staining

Mice from each group were sacrificed by cervical dislocation at 4 days postinoculation (dpi) and 7 dpi. The lung tissues of mice were harvested and fixed in 10% neutral buffered formalin at 4 °C for 5 days. After dehydration in a graded ethanol series with increasing concentration (70, 80, 90, and 100%), the lung tissues were processed by paraffin embedding and were then sectioned and stained. Serial tissue sections (4 μm) were deparaffinized by xylene for 5 min, and the xylene was removed with a graded ethanol series of decreasing concentration (100, 95, 90, 80, and 70%). Lung tissues were rinsed with double distilled water for 3 min, stained in haematoxylin for 10 min, dehydrated in a graded ethanol series with increasing concentration (70, 80, and 90%), stained with eosin for 3 s and dehydrated in a graded ethanol series with decreasing concentration (100 and 95%) for 3 min. The section was covered with Permount TM Mounting Medium and mounted with a coverslip, which was followed by the examination of tissue sections with an optical microscope (Olympus).

### RNA isolation

Total RNA was extracted from 100 mg lung tissues from each mouse according to the manufacturer’s protocol and examined with a Thermo Scientific™ Nanodrop 2000 spectrophotometer. RNA integrity number (RIN) was calculated with a Bioanalyzer 2100 (Agilent Technologies). The OD 260/280 ratios of the samples were maintained between 1.8 and 2.2. The amount of RNA was maintained at approximately 1 μg. Oligo (dT) magnetic beads were used to isolate mRNA from total RNA. By implementing tru-Seq mRNA library (Illumina) purification, total RNA was used as the input for oligo-dT purification to purify the mRNA. Integrated mRNAs have an average length of several thousand- base pairs and cannot be measured by Illumina platform. mRNAs were cleaved to approximately 300 bp randomly in fragmentation buffer and reverse-transcribed to cDNA by random hexamers and these reactions were catalysed with reverse transcriptase. The cohesive ends of the cDNAs were converted into blunt ends by using End Repair Mix (Qiagen).

### miRNA high-throughput sequencing analysis

After reverse transcription, cDNAs were enriched by PCR for 11–12 cycles and purified by 6% Novex TBE PAGE gels (Invitrogen), followed by the addition of TBS380 (Picogreen) to the cDNAs. Clusters were obtained with a cBot (Illumina) by PCR amplification and sequenced by the Illumina HiSeq platform. Clean reads were obtained by removing low quality tags, cleaning and selecting sequences according to the length in base pairs. The lengths of the cleaned small RNAs and the base preference were analysed. The redundant clean reads that had same sequences were eliminated. The cleaned small RNAs were annotated and classified by comparison with sRNAs (siRNAs, piRNAs, and miRNAs) in Rfam and miRBase. The targets of the miRNAs were predicted by miRNA analysis and PCA analysis.

### mRNA high-throughput sequencing analysis

cDNAs were enriched by PCR for 15 cycles. The objective strips were collected with 2% Certified Low- Range Ultra Agarose (Bio-Rad). Through Picogreen (Invitrogen) technology, RT-activity was evaluated by using TBS380 (Invitrogen). PCR amplification was performed to harvest clusters with the cBot, and the clusters were sequenced by the Hiseq platform (Illumina). The cleaned data were obtained by removing low quality tags, cleaning and selecting sequences according to the length in base pairs and analysed according to the reference genome. The target genes of similarly expressed mRNAs were analysed with expressive pattern cluster analysis.

### Procedure for identifying miRNA-mRNA modulatory relationships by conjoint analysis

Gene Ontology (GO) enrichment analysis (http://www.geneontology.org/) and Kyoto Encyclopedia of Genes and Genomes (KEGG) database analysis (http://www.genome.jp/kegg/) were implemented to analyse the target genes of differentially expressed miRNAs and mRNAs. The method that we used to predicts the human miRNA targets by colligating gene expression data with literature information was performed as follows. First, aberrantly expressed miRNAs and mRNAs induced by FM1 and differentially expressed miRNAs and mRNAs after treatment with XDY in comparison with those in the virus group were surveyed by bioinformatics analysis. Differentially or aberrantly expressed mRNAs might be the predicted targets of miRNAs. Mouse miRNA-mRNA pairs were further screened by determination of the reversed correlations between mRNAs and miRNAs in the second step of the processing of omics data. Finally, the differentially or aberrantly expressed miRNAs and mRNAs were assessed for statistical significance (absolute fold change value ≥1 and *P* ≤ 0.05).

### Quantitative real-time PCR (qRT-PCR)

Total RNA was preserved at 4 °C, diluted at a ratio of 1:40 or 1:100 and quantified. We synthesized cDNA from miRNA and mRNA with the corresponding microRNA-specific stem-loop RT primers and the corresponding mRNA-specific stem-loop RT primers (Table 2). Reverse transcription of miRNAs was performed at 37 °C for 1 h and at 95 °C for 5 min, and the products were preserved at − 20 °C. Reverse transcription of mRNAs was performed at 42 °C for 1 h and 70 °C for 5 min, and the products were preserved at − 20 °C. A volume of 2 μl of cDNA reverse-transcribed from miRNAs and mRNAs was used for qRT-PCR with 6 μl of RNase-free water, 1 μl of each primer and 10 μl of PCR mix. PCR was performed at 94 °C for 3 min followed by 30 cycles of 94 °C for 30 s for template denaturation, 55 °C for 30 s for annealing, and 72 °C for 30 s for extension and a final step at 72 °C for 5 min. The relative quantification of the gene expression was calculated by the 2 ^-∆∆CT^ method (threshold cycle).

**Table 2:** Summary of 11 miRNAs and 5 mRNAs

	Seq-ID	Primers sequence	
miRNA	miR-200b-3p	5′-CCGCGTAATACTGCCTGGTAATGATGA-3′	
	miR-351-5p	5′-TCCCTGAGGAGCCCTTTGAG-3′	
	miR-34c-5p	5′-CGAGGCAGTGTAGTTAGCTGATTGC-3′	
	miR-146b-3p	5′-GCCCTAGGGACTCAGTTCTGG-3′	
	miR-669a-3p	5′-GCGCGACATAACATACACACACACGTAT-3′	
	miR-146b-5p	5′-CCGGTGAGAACTGAATTCCATAGGCT-3′	
	miR-147-3p	5′-GGTGTGCGGAAATGCTTCTGCTA-3’	
	miR-155-5p	5′-CCCGGTTAATGCTAATTGTGATAGGGGT-3’	
	miR-223-3p	5′-CGCTGTCAGTTTGTCAAATACCCCA-3’	
	miR-7b-5p	5′-GGCCTGGAAGACTTGTGATTTTGTTGT-3’	
	miR-503-5p	5′-TAGCAGCGGGAACAGTACTGC-3’	
mRNA	ENSMUSG00000063065 (Mapk3)	forward	5′-CTGCTGGACCGGATGTTAACCTTC-3’
		reverse	5′-ACTGGCTCATCTGTCGGATCGTAG-3’
	ENSMUSG00000046709 (Mapk10)	forward	5′-GGAACTGATGGACGCCAACCTG-3’
		reverse	5′-ACACAGCATCTGGTACAGCAAGTAAG-3’
	ENSMUSG00000021250 (Fos)	forward	5′-ACCGTGTCAGGAGGCAGAGC-3’
		reverse	5′-GCAACGCAGACTTCTCATCTTCAAG-3’
	ENSMUSG00000026104 (Stat1)	forward	5′-TCTCATTGTCACCGAAGAACTTCACTC-3’
		reverse	5′-CCAGCATGTTGTACCACAGGATAGAC-3’
	ENSMUSG00000048806 (Ifnb1)	forward	5′-GATGTCCTCAACTGCTCTCCACTTG-3’
		reverse	5′-CATCCAGGCGTAGCTGTTGTACTTC-3’

## Results

### Amelioration of clinical manifestations and pathological alterations by XDY in FM1-infected mice

**Fig. 1:**
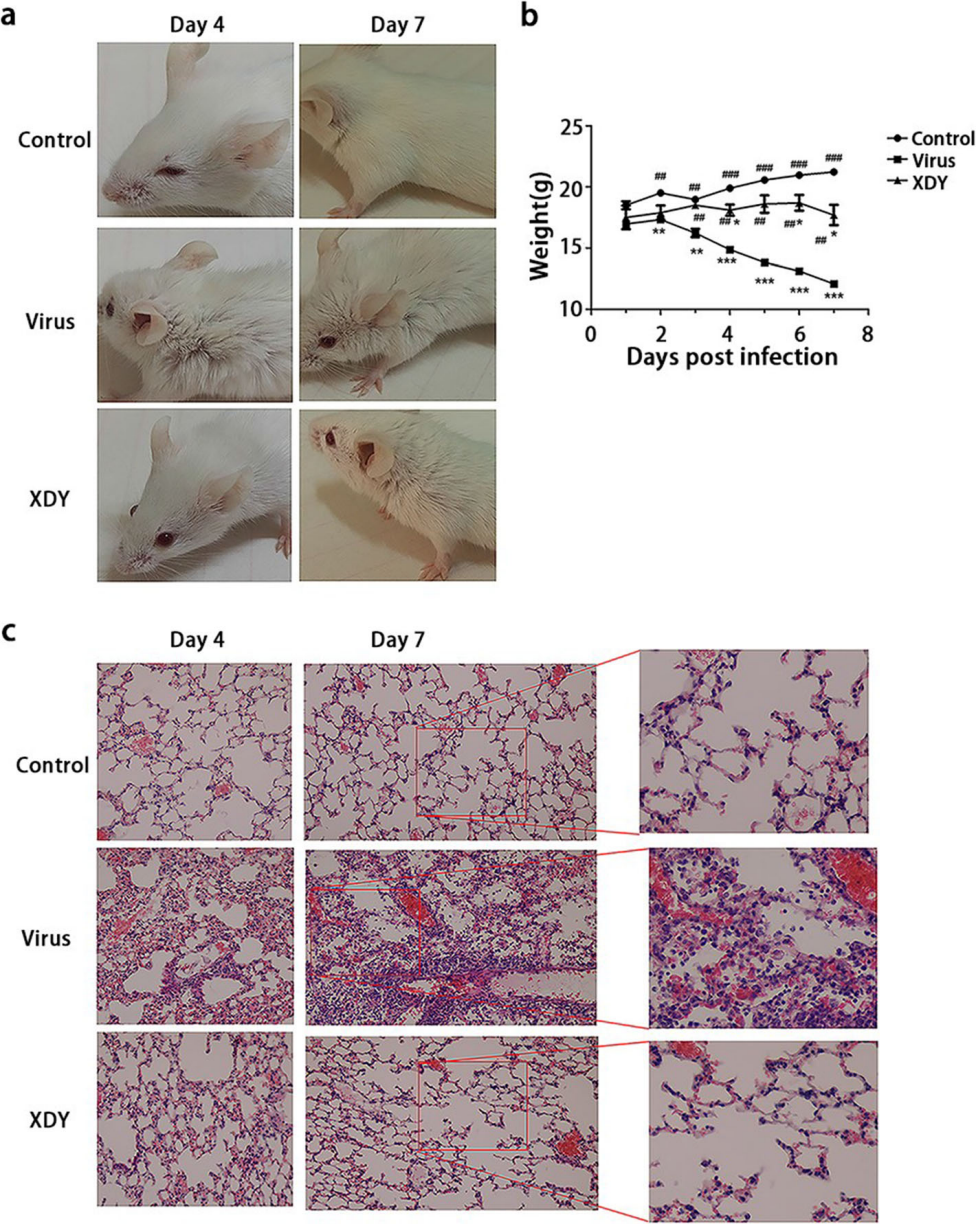
The antiviral pneumonia efficacy of XDY in FM1-infected mice. **a**: Assessment of the appearance and motility changes in the control group, virus group and XDY group at 4 dpi and 7 dpi. **b**: Body weight changes over 7 days in the control group, virus group and XDY group. *differs significantly (*P* ≤ 0.05), ** differs significantly(*P* ≤ 0.01) and ***differs significantly(*P* ≤ 0.001) when compared with the control group. #differs significantly (P ≤ 0.05), ##differs significantly(P ≤ 0.01) and ###differs significantly (P ≤ 0.001) when compared with the virus group. **c**. XDY ameliorated the injury of alveolar structure and the infiltration of inflammatory cells compared with that in the virus group as shown by H&E staining of mouse models

We validated the therapeutic effect of XDY on FM1-induced pneumonia by observing the clinical manifestations and detecting lung histopathologic changes. As described in Fig. 1a, FM1-infected mice showed weakness and more ruffled and rougher fur compared with the control mice. Mice with XDY administration had smoother hair and a faster response and were significantly more hyperactive than the mice in the virus group. FM1-infected mice showed a sharp reduction in body weight at 7 dpi, whereas the change in the weight of XDY-treated mice was not significant in comparison that in noninfected mice (Fig. 1b). (*P* ≤ 0.05).

As shown in Fig. 1c, the histological results revealed pulmonary interstitial inflammation, which was characterized by infiltration of inflammatory cells, hyperaemia and haemorrhage around the blood vessels in lung tissues, as well as thickening of alveolar septa in FM1-infected mice at 4 dpi. At 7 dpi, more severe pathological alterations were observed in infected mice, including pulmonary interstitial deterioration and inflammation, in addition to alveolar collapse. Furthermore, mice administered with XDY exhibited an alleviation of lung histopathology in terms of grade compared with FM1-infected mice.

### The effects of XDY on miRNA expression patterns in response to FM1 infection

**Fig. 2:**
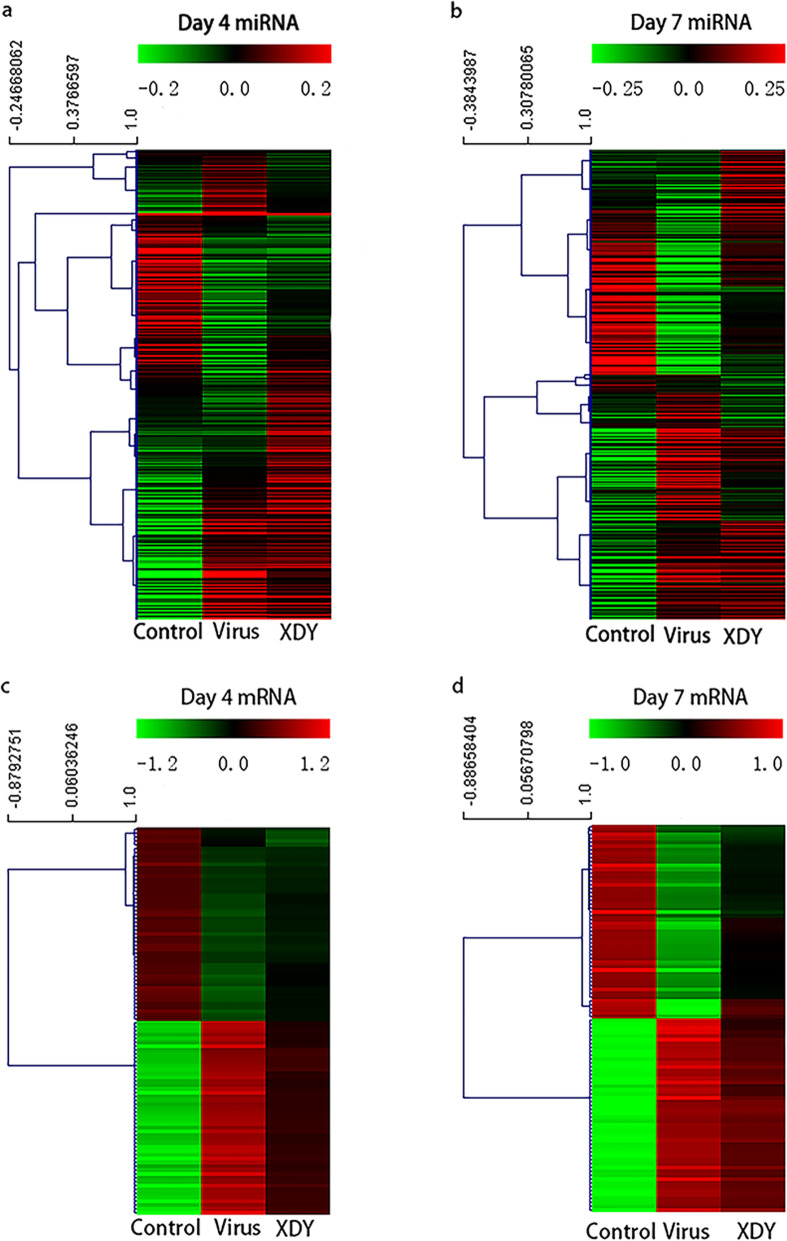
Hierarchical cluster analysis of miRNAs and mRNAs in mouse lungs at 4 dpi and 7 dpi after FM1 challenge. **a**: The expression of miRNAs in the control group, virus group and XDY group at 4 dpi. The colour reveals the level of expression of the miRNA, log10 FPKM. **b**: The expression of miRNAs in the control group, virus group and XDY group at 7 dpi. The colour represents the level of expression of miRNA, log10 FPKM. **c**: The expression of mRNAs in the control group, virus group and XDY group at 4 dpi. The colour reveals the level of expression of the mRNA, log10 FPKM. **d**: The expression of mRNAs in the control group, virus group and XDY group at 7 dpi. The colour represents the level of expression of miRNA, log10 FPKM. Red and green colours indicate the high and low expression of miRNAs

**Fig. 3:**
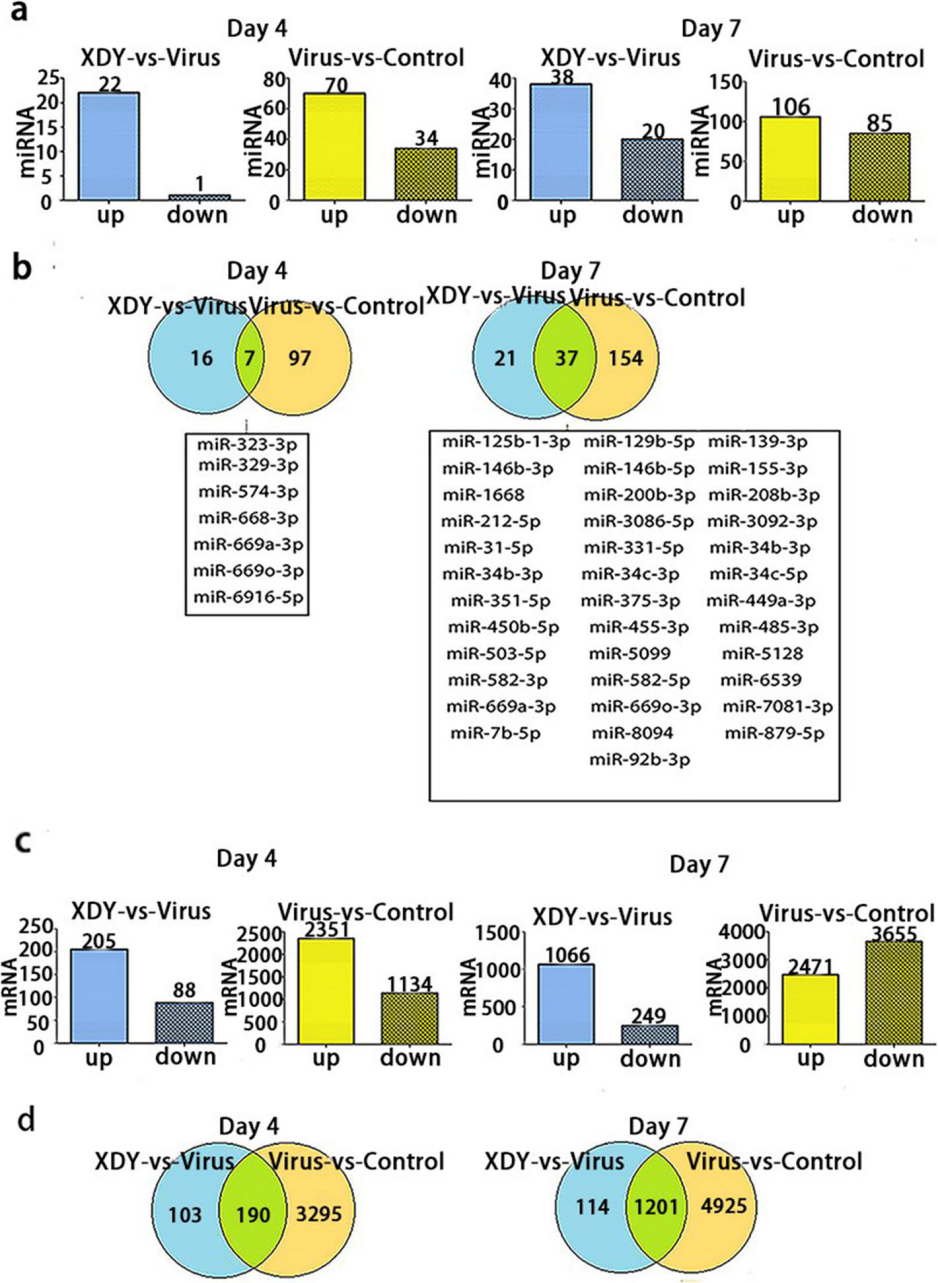
Differentially expressed miRNAs and mRNAs in XDY-vs-Virus and Virus-vs-Control at 4 dpi and 7 dpi. **a**: The quantity of differentially expressed miRNAs in XDY-vs-virus and virus-vs-control groups at 4 dpi and 7 dpi. **b**: Comparison of differentially expressed miRNAs between XDY-VS-virus and virus-vs-control at 4 dpi and 7 dpi. **c**: The quantity of differentially expressed mRNAs in XDY-vs-virus and virus-vs-control groups at 4 dpi and 7 dpi. **d**: Comparison of differentially expressed mRNAs between XDY-vs-virus and virus-vs-control at 4 dpi and 7 dpi

To explore the mechanisms of the effects of XDY on miRNAs in FM1-infected mice, we carried out cellular miRNA profiling in response to FM1 infection and XDY treatment through high-throughput sequence analysis. Among the 1308 cellular miRNAs identified by high-throughput sequencing, 364 known miRNAs were detected in lung tissues, as shown in Fig. 2a and b. As illustrated in Fig. 3a, 104 miRNAs were significantly changed in mice with FM1 infection at 4 dpi, of which 70 miRNAs were upregulated and 34 miRNAs were downregulated. At 7 dpi, a total of 191 miRNAs exhibited differential expression in mice with FM1 infection, of which 106 miRNAs were upregulated and 85 miRNAs were downregulated. After XDY treatment, the expression of 22 miRNAs were increased at 4 dpi, while the expression of 1 miRNA was decreased. At 7 dpi, the number of differentially expressed miRNAs increased to 58, among which, 20 miRNAs were downregulated and 38 miRNAs were upregulated. (|fold change| ≥1 and *P* ≤ 0.05).

The overlapping miRNAs, which were differentially expressed in both the virus-vs-control group and the virus-vs-XDY group, are displayed in Fig. 3b. In total, there were 113 miRNAs that exhibited distinct differential expression at 4 dpi and 175 miRNAs that showed differential expression at 7 dpi. Venn diagrams also indicated that there were 7 and 37 common differentially expressed miRNAs at 4 dpi and 7 dpi, respectively. The number of distinct or common differentially expressed miRNAs increased over time.

### The effects of XDY on the mRNA expression pattern in response to FM1 infection

Among the 47,729 mRNAs identified by high-throughput sequencing, a total of 7961 mRNAs were detected in lung tissues, and 100 mRNAs identified at 4 dpi and 7 dpi are shown in Fig. 2c and d, respectively. As depicted in Fig. 3c, 3485 mRNAs exhibited significant differential expression in FM1 infection at 4 dpi, of which 2351 mRNAs were upregulated and 1134 mRNAs were downregulated. At 7 dpi, the number of differentially expressed mRNAs dramatically increased to 6126, of which 2471 mRNAs were upregulated and 3655 mRNAs were downregulated. After XDY treatment, 293 mRNAs were significantly changed at 4 dpi, of which 205 miRNAs were upregulated and 88 miRNAs were downregulated. At 7 dpi, 1315 mRNAs exhibited distinct differential expression after treatment with XDY, of which 1066 mRNAs were upregulated and 249 mRNAs were downregulated. In total, the number of differentially expressed mRNAs increased over time. (|fold change| ≥1 and *P* ≤ 0.05).

The differentially expressed mRNAs between the FM1 infection and XDY treatment groups were compared. The distinct and common differentially expressed mRNAs between the XDY-vs-virus and the virus-vs-control groups are described in Fig. 3d. In total, there were 3398 mRNAs that exhibited distinct differential expression at 4 dpi and 5039 mRNAs that exhibited distinct differential expression at 7 dpi. Venn diagrams revealed 190 common differentially expressed mRNAs at 4 dpi and 1201 common differentially expressed mRNAs at 7 dpi.

### FM1 infection and XDY treatment-associated miRNA–mRNA network

To explore the miRNA-mRNA network generated in response to pneumonia caused by FM1 infection and XDY supplementation, comprehensive analysis of interacting miRNA and mRNA was performed according to 3 criteria: 1. the differentially expressed mRNAs must be the predicted targets of miRNAs; 2. the mRNAs were inversely correlated with the miRNAs; and 3. the differentially expressed miRNAs and mRNAs showed statistical significance (absolute fold change value ≥1 and *P* ≤ 0.05).

**Fig. 4:**
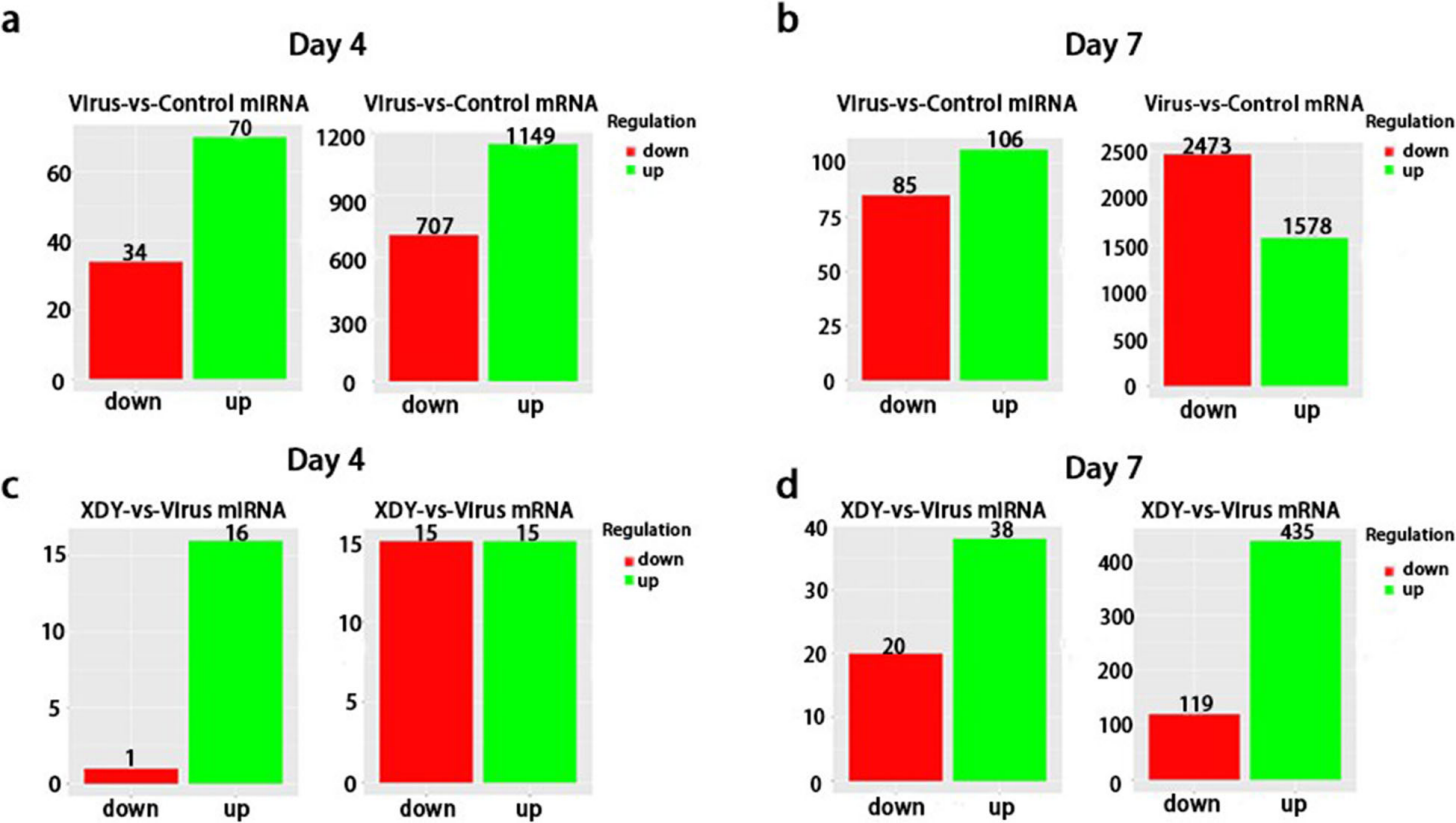
Differentially expressed miRNAs and mRNAs after miRNA-mRNA comprehensive analysis. **a**: The quantity of differentially expressed miRNAs and correlative mRNAs in the virus-vs-control group at 4 dpi. **b**: The quantity of differentially expressed miRNAs and correlative mRNAs in the virus-vs-control group at 7 dpi. **c**: The quantity of differentially expressed miRNAs and correlative mRNAs in the XDY-vs-virus group at 4 dpi. **d**: The quantity of differentially expressed miRNAs and correlative mRNAs in the XDY-vs-virus group at 7 dpi

In FM1-infected lungs, 70 upregulated and 34 downregulated miRNAs matched 707 downregulated and 1149 upregulated mRNAs at 4 dpi, respectively. After XDY treatment, 1 downregulated and 16 upregulated miRNAs matched 15 upregulated and 15 downregulated mRNAs, respectively. In FM1-infected lungs at 7 dpi, a total of 106 upregulated miRNAs matched 2473 downregulated mRNAs, and 85 downregulated miRNAs matched 1578 upregulated mRNAs. In XDY-treated lungs, a total of 38 upregulated miRNAs that matched with 119 inversely expressed mRNAs and 20 downregulated miRNAs that matched with 435 upregulated mRNAs were identified (Fig. 4 and Table S1).

### Gene ontology (GO) and Kyoto encyclopedia of genes and genomes (KEGG) pathway analyses

**Fig. 5:**
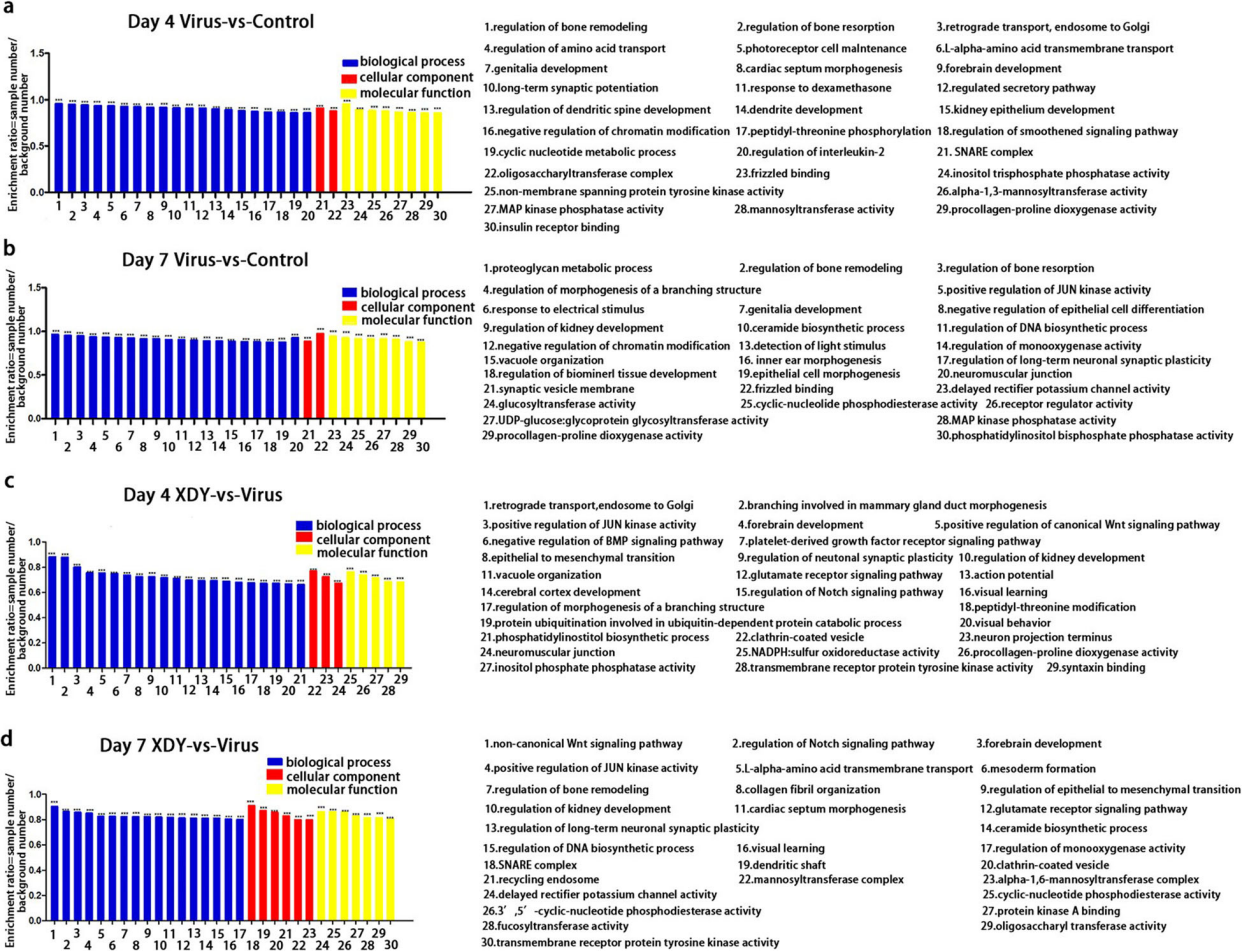
GO enrichment analysis of differentially expressed target genes. **a**: Virus-vs-control lungs at 4 dpi. **b**: virus-vs-control lungs at 7 dpi. **c**: XDY-vs-virus lungs at 4 dpi. **d**: XDY-vs-virus at 7 dpi. GO terms are exhibited by the abscissae, and the enrichment ratios (sample number/background number) of candidate target genes annotated to this term are exhibited by the ordinates. *differs significantly (P ≤ 0.05), ** differs significantly(P ≤ 0.01) and ***differs significantly(P ≤ 0.001)

To further elucidate how miRNAs change in response to XDY treatment of FM1 infection in terms of potential biological processes and signalling pathways, GO and KEGG pathway analyses were performed. A total of 30, 554, 1856 and 4051 identified target mRNAs, which were acquired from XDY-treated and FM1-infected mice at 4 dpi and 7 dpi, were annotated and assigned to GO categories, including biological process (BP), cellular component (CC) and molecular function (MF) categories. Figure 5 shows the top 30 enriched GO entries of associated with virus-infected lungs and XDY-treated lungs at 4 dpi and 7 dpi. Among the three GO categories, the most enriched targets were associated with BP. The top 5 enriched BP terms were regulation of JUN kinase (JNK) activity, regulation of IL-2 production, proteoglycan metabolic process, L-alpha-amino acid transmembrane transport and regulation of the DNA biosynthetic process in FM1-infected lungs, while regulation of JNK activity, L-alpha-amino acid transmembrane transport, regulation of the DNA biosynthetic process, platelet-derived growth factor receptor signalling pathway and regulation of monooxygenase activity were associated with the treatment of XDY (*P* ≤ 0.05). The top 3 enriched MF terms associated with FM1 infection were with MAPK phosphatase activity, inositol trisphosphate phosphatase activity and procollagen-proline dioxygenase activity, while protein kinase A (PKA) binding, cyclic-nucleotide phosphodiesterase (CNP) activity and transmembrane receptor protein tyrosine kinase activity were linked to XDY-treated lungs (P ≤ 0.05).

**Fig. 6:**
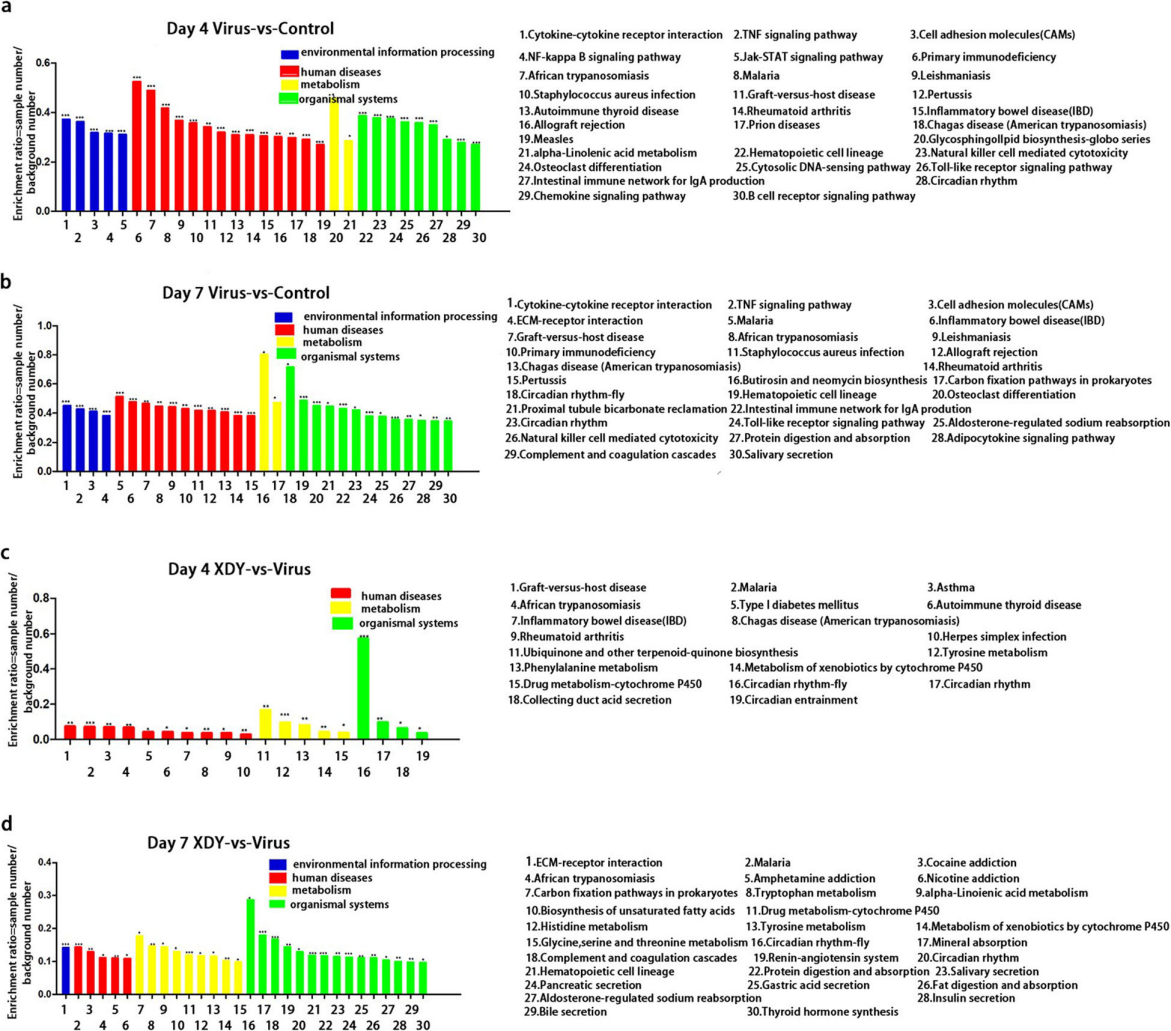
KEGG pathway classifications of differentially expressed target genes. **a**: Virus-vs-control lungs at 4 dpi. **b**: virus-vs-control lungs at 7 dpi. **c**: XDY-vs-virus lungs at 4 dpi. **d**: XDY-vs-virus at 7 dpi. The stacked bars denote the top 30 KEGG pathways in virus-vs-control lungs at 4 dpi and 7 dpi, XDY-vs-virus lungs at 7 dpi and the top 19 KEGG pathways in virus-vs-control lungs at 4 dpi. *differs significantly (P ≤ 0.05), ** differs significantly(P ≤ 0.01) and ***differs significantly(P ≤ 0.001)

The KEGG analysis indicated that the differentially expressed miRNAs, which were conversely correlated with mRNAs, were mainly associated with the following 10 pathways after FM1 infection: TLR signaling pathway, Janus kinase-Signal transducer and activator of transcription (Jak-STAT) signaling pathway, TNF signalling pathway, natural killer (NK) cell- mediated cytotoxicity, haematopoietic cell lineage, NF-kappa B (NF-κB) signalling pathway, circadian rhythm-fly, extra cellular matrix (ECM)-receptor interaction, cell adhesion, and the adenosine monophosphate-activated protein kinase (AMPK) signalling pathway (Fig. 6). The differentially expressed miRNAs, in XDY treated mice in response to FM1-infection, were mainly involved in 7 pathways: complement and coagulation cascades, circadian rhythm-fly, drug metabolism-cytochrome 450, histidine metabolism, tyrosine metabolism, extracellular matrix (ECM)-receptor interaction, and glycine, serine and threonine metabolism (Fig. 6).

### Validation of miRNA and mRNA expression by qRT-PCR

qRT-PCR was performed to verify the expression of miRNA and mRNA as quantified by high-throughput sequencing. According to the comprehensive miRNA–mRNA analysis and the GO and KEGG pathway analyses, the expression of 11 significantly differentially expressed miRNAs, miR-146b-3p, miR-146b-5p, miR-223-3p, miR-200b-3p, miR-34c-5p, miR-351-5p, miR-503-5p, miR-147-3p, miR-155-5p, miR-669a-3p, and miR-7b-5p, and 5 mRNAs, Mapk3, Mapk10, Fos, Stat1 and Interferon-β1 (Ifnb1), were selected and verified by qRT-PCR.

**Fig. 7:**
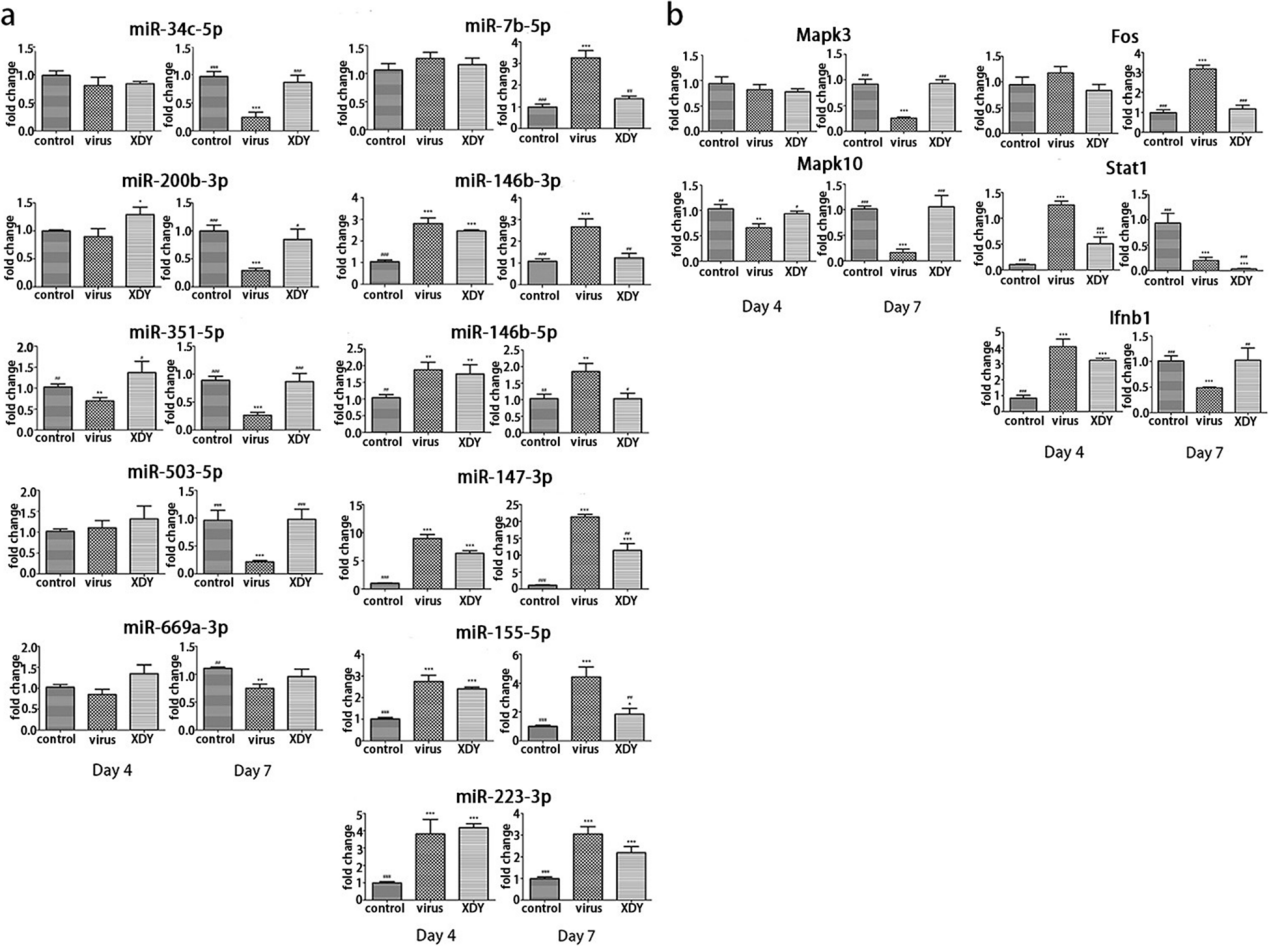
qRT-qPCR verification for some differentially expressed miRNAs and mRNAs. **a**. The expression of 11 miRNAs in virus-vs-control lungs, XDY-vs-virus lungs at 4 dpi and 7 dpi. **b**. The expression of 5 mRNAs in virus-vs-control lungs, XDY-vs-virus lungs at 4 dpi and 7 dpi. *differs significantly (P ≤ 0.05), ** differs significantly(P ≤ 0.01) and ***differs significantly(P ≤ 0.001) when compared with the control group. #differs significantly (P ≤ 0.05), ##differs significantly(P<0.01) and ###differs significantly (P ≤ 0.001) when compared with the virus group

Generally, the results for the differentially expressed miRNAs and mRNAs exhibited good consistency with the high-throughput sequencing results, as demonstrated by qRT-PCR. In the larger cohort, 6 miRNAs (miR-7b-5p, miR-146b-3p, miR-146b-5p, miR-147-3p, miR-155-5p and miR-223-3p) of the 11 miRNAs increased after challenge with FM1, while 4 miRNAs (miR-200b-3p, miR-34c-5p, miR-351-5p and miR-669a-3p) were suppressed. MiR-503-5p was slightly upregulated at 4 dpi after FM1 incubation, but was suppressed at 7 dpi. The qRT-PCR assay revealed the upregulation of miR-34c-5p, miR-200b-3p, miR-351-5p, miR-669a-3p and miR-503-5p in XDY-treated mice, whereas 5 miRNAs (miR-7b-5p, miR-146b-3p, miR-146b-5p, miR-147-3p, and miR-155-5p) were downregulated. MiR-223-3p was slightly upregulated by XDY supplementation at 4 dpi, but suppressed at 7 dpi (Fig. 7a). The results showed a decrease in Mapk3 mRNA caused by FM1 infection at both time points, and Mapk3 mRNA was downregulated by XDY administration at 4 dpi, but was upregulated at 7 dpi. Mapk10 mRNA was downregulated after infection with FM1 on both time points, while upregulated by XDY supplementation. Fos mRNA was upregulated after FM1-infection, and downregulated after XDY treatment. The Stat1 and Ifnb1 mRNAs were upregulated in FM1-infected mice at 4 dpi, while they were downregulated at 7 dpi. XDY treatment inhibited Stat1 and Ifnb1 mRNA expression at 4 dpi, but enhanced Ifnb1 mRNA expression at 7 dpi (Fig. 7b). (*P* ≤ 0.05).

## Discussion

### IAV infection induced aberrant miRNA expression profiles and XDY treatment modulated the miRNA expression patterns

IAV-induced miRNA expression patterns were temporally dependent- and strain-specific [4]. Routinely, the quantity of differentially expressed miRNAs triggered by IAV increased over time. Our results showed that the number of differentially expressed miRNAs increased in FM1-induced pneumonic mice from 104 at 4 dpi to 191 at 7 dpi. The expression patterns of miRNAs after IAV challenge rely greatly on virus strains, as indicated by the evidence gained from studies that compared differences in the miRome in response to different IAV strains with differences in pathogenicity or host background. We identified some differentially expressed miRNAs during infection with various IAVs in mice or pigs, as shown in Table 3, demonstrating the common or distinct miRNA responses in animal lungs after IAV challenge [10, 13, 37–43]. MiR-151-5p and miR-223-3p, which were commonly upregulated during ma81 or w81 infection to varying degrees, were indicated to be responsible for contributing to the pathogenesis of flu [13]. We also found that the level of miR-223-3p increased after FM1 challenge, while miR-151-5p was inversely expressed, suggesting that FM1 shares some virulence characteristics with ma81 or w81 as well as some differential pathogenic mechanisms based on the miRome. FM1 could induce the upregulation of miR-7, miR-221-5p, miR-223-5p, and miR-146a-5p in the lungs of mice, which was also observed in A/sw/Denmark/12687/03 (H1N2) infected pig lungs, while miR-223-5p was inversely expressed [39]. Moreover, the miRNA profile after FM1 challenge shared few similarities with that of H1N2, which could be explained by the difference between the virus strains and host background. Additionally, there were some specific FM1-induced miRNAs, such as miR-351-5p, miR-669a-3p, miR-200b-3p, miR-212-5p, miR-125b-1-3p, and miR-34c-5p. The importance of miRNAs in regulating IAV infection and the host antiviral response has been validated, and major studies have focused on differentially expressed miRNAs. Our study first provided information on pulmonary miRNA profiles induced by FM1 and further demonstrated the virus strain- and host-specific pattern of the miRome after IAV challenge. We assumed that some common differential miRNAs might play important roles during IAV infection, since they were conservatively utilized by various IAVs to invade host defence. The distinct differentially expressed miRNAs during the infection course of different IAV strains might be correlated with differences in pathogenicity. Thus, in future studies, we will explore the function of some common differentially expressed miRNAs, such as miR-146b-3p, miR-155-3p, and miR-223-3p, in FM1-infected mice to further demonstrate the identical characteristics of the host-IAV interaction. We will also need to investigate some special differentially expressed miRNAs, such as miR-221-5p and miR-212-5p to reveal the potential roles of these miRNAs in FM1 pathogenicity and host defence.

**Table 3:** The expression of pulmonary miRNAs in animals infected with different IAV strains

miRNA	A/Aquatic bird/ Korea/ma81/2007 (H5N2)	A/Aquatic bird/Korea/w81/2005 (H5N2)	A/Fort Monmouth/1/47 (H1N1)	A/sw/Denmark/12687/03 (H1N2)
miR-100-5p	Up	Up	Down	
miR-146b-3p	Up	Up	Up	
miR-130a-5p	Up	Up	Up	
miR-151-5p	Up	Up	Down	
miR-147-3p		Up	Up	
miR-155-3p	Up	Up	Up	
miR-223-3p	Up	Up	Up	Up
miR-223-5p			Up	Up
miR-495-3p	Up	Up	Down	
miR-351-5p	Up	Up	Down	
miR-221-5p			Up	Up
miR-669a-3p			Down	
miR-200b-3p			Down	
miR-7			Up	Up
miR-146a-5p			Up	Down
miR-125b-1-3p			Up	
miR-34c-5p			Down	
miR-212-5p			Up	
miR-21			Up	

Liu et al. found that 23 active compounds obtained from XJDHD might interact with 118 target genes involved in the NF-κB, AMPK and PI3K-AKT pathways, to prohibit excessive inflammatory response and repair the circulatory system during viral haemorrhagic fevers [44]. Our previous study showed that XDY inhibited influenza-induced F-actin reorganization in PMVECs by suppressing the activation of the Rho/ROCK and PKC signalling pathways as well as the phosphorylation of myosin light chain (p-MLC) [34]. In this study, we found that XDY inversely regulated aberrant miRNA expression induced by FM1 challenge. For example, the upregulation of miR-146b-5p, miR-212-5p, miR-7b-5p, and miR-6539 and the downregulation of miR-872-3p, miR-200b-3p, miR-34b-5p, miR-34b-3p, miR-34c-5p, and miR-3086-5p during FM1 infection were inversely altered by XDY. Based on the GO/KEGG enrichment analysis, 11 miRNAs (miR-146b-3p, miR-146b-5p, miR-223-3p, miR-200b-3p, miR-34c-5p, miR-351-5p, miR-503-5p, miR-147-3p, miR-155-5p, miR-669a-3p, and miR-7b-5p) that might be associated with Jak-STAT, MAPK phosphatase activity, TLR, RIG-I-like receptor and the NOD-like receptor signalling pathways were selected for qPCR validation.

Li et al. described that upregulation of miR-7b was involved in anti-fibrotic function by repressing the activation of JNK, TGF-β, extracellular signal-regulated kinase (ERK), and p38 in cardiac myocytes [45]. Our results showed a rapid increase in miR-7b-5p after FM1 infection, but a decrease after treatment with XDY. Choi EJ et al. demonstrated that miR-223-3p was upregulated after infection with w81 and ma81 and enhanced the differentiation of granulocytes [13]. Our study also found a comparable result: miR-223-3p increased during FM1 infection, but decreased after XDY treatment. The results for the tendencies of the expression of miR-7b-5p and miR-223-3p were consistent with those of the studies by Li and Choi EJ. Amaral AJ et al. indicated that miR-34c-5p was upregulated after CK/TX/02/H5N3 avian IAV or HIV challenge [46, 47]. Our work suggested that miR-34c-5p, whose targets might be the Stat3 and Prkcb mRNAs, was decreased by FM1 infection, but was upregulated after treatment with XDY. The expression data for miR-34c-5p in our study was inconsistent with that of Amaral AJ, which further confirmed the viral strain-specific characteristic of miRNA expression. Similar to that of miR-34c-5p, the expression tendency of miR-200b was also virus strain-specific. MiR-200b decreased in mouse lungs after infection with r1918 H1N1, but increased after challenge with A/Texas/36/91 H1N1, which was correlated with the acceleration of the prostaglandin biosynthetic process [4]. Our work showed that miR-200b-3p, whose target might be Stat1 mRNA, was downregulated after infection with FM1, but was upregulated after XDY treatment.

Infected with CK/TX/02/H5N3 avian influenza, reductive miR-146b enhanced the expression of IL-1 receptor associated kinase 1 (IRAK1) and TNF receptor-associated factor 6 (TRAF6) [47]. Tomita et al. described that upregulated miR-155 suppressed activator protein-1 (AP-1) activation by depressing the expression of BTB and CNC homology 1 (BACH1) and restrained the development of human T-cell leukaemia virus type 1 (HTLV-1) -positive T-cell lines [48]. Our work showed that miR-146b-3p, miR-146b-5p and miR-155-5p increased during FM1 infection and decreased by XDY treatment, and the variation tendencies of miR-146b-3p, miR-146b-5p and miR-155-5p after IAV challenge were consistent with Tomita’s study. Our work indicated that miR-147-3p increased with FM1 infection, but decreased dramatically with XDY supplementation. Mapk10 mRNA might be the target of miR-155-5p, while no mRNA targets of miR-146b and miR-147-3p were suggested in our study. We analysed the potential reasons due to the criterion of the miRNA-mRNA comprehensive analysis, which claimed that targeted mRNA variations should be inversely correlated with miRNA variations, while the miR-146b and miR-147-3p target genes might not fit the criterion. The miRNA-mRNA networks in lungs presenting the synthetic effects of all cell types in tissues, showcased more intricate characteristics compared with the miRNA-mRNA profiles in a single cell line.

Previous studies indicated that miR-503-5p, miR-351-5p and miR-669a-3p might participate in the processes of atherosclerosis and oesophageal squamous cell carcinoma [49, 50]. Our results showed that the expression of miR-503-5p, whose target might be Stat2 mRNA, was upregulated after challenge with FM1 at 7 dpi but decreased by the addition of XDY. MiR-351-5p, whose target might be Stat1, Stat2, Stat3, pycard and Myd88 mRNAs, was downregulated with FM1, but increased by XDY. MiR-669a-3p, targeting Stat3 mRNA, decreased with FM1 infection, and increased with XDY supplementation. Accordingly, this is the first report of miR-503-5p, miR-351-5p and miR-669a-3p variations after IAV challenge. Further studies are warranted to explore their elaborate roles in FM1 pathogenesis or immunologic response.

As the first water channel to be identified, Aqp1 not only transports water across cytomembranes, but also participates in cytoskeleton catenin, build-up and motility [51–53]. However, the role of Aqp1 and the miRNAs regulating its expression has not been reported. Interestingly, our study identified multiple miRNAs, such as miR-223-3p, miR-6539, miR-7a-5p, and miR-18b-3p, targeting Aqp1 mRNA, which suggested that Aqp1 might play a role in IAV infection and needs to be further explored, which warrants further investigation.

### IAV infection induced an aberrant mRNA expression profile, and XDY treatment modulated the mRNA expression patterns

IAV infection triggers activation of TLR, PKC, MAPK and inflammasome pathways, leading to production of proinflammatory cytokines. Many traditional Chinese medicine formulas, such as San Wu Huangqin Decoction, Gegen Qinlian Decoction, and Ma Huang Tang have been indicated to have protective effects on IAV-infected mice by modulating the TLR pathway to maintain a balanced inflammatory response [54–56]. Previous studies have indicated that XDY could inhibit the overreaction of TLR, PKC and inflammasome pathways to exert therapeutic efficacy on viral pneumonia induced by IAV challenge [30–32, 34]. However, the effects of XDY on the ERK, JNK, AP-1, and Jak-STAT pathways have not been elaborated during the FM1 infection course. Thus, the levels of Mapk3, Mapk10, Fos, Stat1 and Ifnb1 mRNAs were validated by qRT-PCR in our study.

We noted that Mapk3 (Erk1) mRNA and Mapk10 (Jnk3) mRNA were reduced during FM1 infection, but enhanced with XDY treatment. Previous studies indicated that p38 MAPK phosphorylation was induced by avian H9N2 Quail/HK/G1/97 (H9N2/G1) and PR8 virus [57, 58]. Kim et al. showed that NF-κB was activated by p-MAPK, IL-1β, and interferon-γ (IFN-γ). The activated MAPK pathway participates in the cellular innate antiviral response, while excessive expression of the MAPK pathway causes host-damaging inflammation [58, 59]. It has been demonstrated that IAV triggers MAPK-dependent phosphorylation of ERK and JNK in the lungs of mice. Thus, we speculated that suppression of Mapk3 and Mapk10 mRNA expression after FM1 infection might be a negative feedback from infected host cells to ERK and JNK pathways. Since the activation of ERK and JNK in the MAPK pathway both regulated IAV infection and caused inflammatory tissue damage if overactivated, XDY could strike the balance between antiviral responses and inflammatory injuries.

C-fos, combined with c-jun, binds to AP-1 DNA sequences and modulates gene expression. AP-1 is essential for IFN-β synthesis, cellular survival and proliferation [60, 61]. AP-1 can be induced by IAV-stimulated SAPK/ERK kinase (SEK)/MPK, MPK kinase 4 (MKK4), MPK and MPK kinase 7 (MKK7) [62]. Inflammatory injuries might be incurred with excessive expression of IFN-β, IL-6 and TNF-α through the activation of AP-1 [62–64]. We found that Fos mRNA increased with FM1 infection, but decreased with XDY treatment. Thus, XDY might ameliorate immunopathology by suppressing Fos mRNA.

*Galani* et al. *indicated that the level of IFN-α increased in mice incubated with 100 pfu (50% lethal dose) PR8 within 5 days and then decreased after 5 days, while the content of IFN-β was not significantly altered* [65]*. Interestingly, in our study, Ifnb1 mRNA significantly increased at 4 dpi and repressed at 7 dpi, which was consistent with the change in IFN-α in Galani’s study, suggesting that IFN-β might exert crucial effects as well as IFN-α in the antiviral response induced by FM1. The “yin-yang” of the type I IFN system has been elucidated as having the robust antiviral effects as well as sequent inflammatory pathogenesis. Thus, after XDY treatment, the reductive Ifnb1 mRNA at 4 dpi might ameliorate inflammatory injuries, while the enhanced Ifnb1 mRNA at 7 dpi might improve antiviral defence. The JAK/STAT signalling pathway, triggered by IFN-α, IFN-γ, IL-6, IL-12, IL-2, erythropoietin (EPO), and IL-4, is crucial in the regulation of the immune response, cell growth, proliferation, survival differentiation, germ stem cell development and haematopoiesis. We found that the variation tendency of Stat1 mRNA, consistent with Ifnb1, was upregulated at 4 dpi, but significantly decreased at 7 dpi after FM1 challenge. Stat1 transcription was depressed by XDY at both timepoints. Type I IFNs (mainly IFN-α and IFN-β) and type III IFNs (IL-29, IL-28a, and IL-28b) are upregulated by IAV and then phosphorylate Jak1, Tyk2, Stat1, and Stat2 to exert a broad range of antiviral immune functions by driving the expression of interferon stimulated genes (ISGs)* [66, 67]*. Additionally, type II IFN (IFN- γ), bound to the IFN-γ receptor (IFNGR), led to Stat1 phosphorylation by the activation of Jak1, Jak2, and IFNGR. Activated Stat1 promoted the recruitment and infiltration of immune cells and mediated broad immune responses to pathogens* [68, 69]*. However, both type I IFNs and type II IFN could lead to collateral damage and immunopathology in the following inflammatory process. Thus, we speculated that XDY could alleviate the detrimental effects of antiviral defence by downregulating Stat1 mRNA levels during IAV infection.*

## Conclusion

In the lung tissues of FM1-infected pneumonic mice, a large number of miRNAs and mRNAs were differentially expressed. Our study indicated that XDY might modulate the expression of miR-146b-3p, miR-146b-5p, miR-223-3p, miR-200b-3p, miR-34c-5p, miR-351-5p, miR-503-5p, miR-147-3p, miR-155-5p, miR-669a-3p, miR-7b-5p and the Mapk3, Mapk10, Fos, Stat1, and Ifnb1 mRNAs, which are associated with the ERK/JNK-AP-1 and IFN-β/STAT signalling pathways, to exert therapeutic effects on FM1-induced pneumonia. Taken together, these results, suggest that the therapeutic mechanisms of XDY may be correlated with regulating the antiviral response and ameliorating excessive inflammatory responses.

## Supplementary information

**Additional file 1.**

## Data Availability

The datasets used and/or analysed during the current study available from the corresponding author opon reasonable request.

## Abbreviations

miRNA: MicroRNA
XDY: Xijiao Dihuang decoction combined with Yinqiao powder
IAV: Influenza A virus
HUVEC: Human umbilical vein endothelial cell
EC: Endothelial cell
r1918: Reconstructed 1918 influenza virus
CREB: cAMP-response Element-binding Protein
type I IFN: Type I interferon
TGF-β: Transforming growth factor-β
RIN: RNA integrity number
GO: Gene Ontology
KEGG: Kyoto Encyclopedia of Genes and Genomes
qRT-PCR: Quantitative real-time PCR
BP: Biological process
CC: Cellular component
MF: Molecular function
TLR: Toll-like receptor
Jak-STAT: Janus kinase-Signal transducer and activator of transcription
TNF: Tumour Necrosis Factor
NK: Natural killer
NF-κB: NF-kappa B
ECM: Extra cellular matrix
AMPK: Adenosine Monophosphate Activated Protein Kinase
DC-STAMP: Dendritic cell-specific transmembrane protein
IBV: Infectious bronchitis virus
IRAK1: IL-1 receptor associated kinase 1
TRAF6: TNF receptor-associated factor 6
ER: Endoplasmic reticulum
HTLV-1: Human T-cell leukaemia virus type 1
BACH1: BTB and CNC homology 1
HIVEP2: Human immunodeficiency virus type 1 enhancer binding protein 2
JEV: Japanese encephalitis virus
SHIP1: Src homology 2-containing inositol phosphatase 1
PRRSV: Porcine Reproductive and Respiratory Syndrome Virus
ATL: Adult T cell leukaemia/lymphoma
PMVEC: Pulmonary microvascular endothelial cell
OA: Osteoarthritis
HDAC: Histone deacetylase
EC: Endothelial cell
HUVEC: Human umbilical vein endothelial cell
HBE: Human bronchial epithelial cell
FUdR: 5-Fluoro-2′-deoxyuridine
PKC: Protein kinase C
AQP-1: Aquaporin-1
Mapk3: Mitogen-activated protein kinase3
ERK: Extracellular signal-regulated kinase
JNK: c-Jun N-terminal kinase
IL-1β: Interleukin 1-β
IFN-γ: Interferon-γ
ALI: Acute lung injury
MKK4: MPK and MPK kinase4
MKK7: MPK and MPK kinase7
ISG: IFN-stimulated gene
CAEV: Caprine arthritis-encephalitis virus
LTR: Long terminal repeat
M-CSF: Macrophage colony-stimulating factor
RANKL: Receptor activator of NF-κB ligand
PVN: Paraventricular nucleus
SON: Supraoptic nucleus
NOD: Nucleotide oligomerization domain
NLRP3: Nucleotide oligomerization domain (NOD)- like receptor pyrin domain-containing 3
ICAM-1: Intercellular adhesion molecule-1
VCAM-1: Vascular cell adhesion molecule-1
GRK2: G protein coupled receptor kinase 2
β-AR: β-adrenergic receptors
GSα: G protein α
p-ERM: Phosphorylation of ezrin/radixin/moesin
MCP-1: Monocyte chemoattractant protein-1
PGE1: Prostaglandin E-1
PLA2: Phospholipase A2
LTB4: Leukotriene B4
ROCK: Rho kinase 1
EPO: Erythropoietin
